# Practical Remarks on the Diseases Which Occurred on Board the Astrea, &c.

**Published:** 1799-05

**Authors:** Stewart Henderson


### *Practical Remarks on the Diseases which occurred on board the Astrea, &c.*—*By* Stewart Henderson.

[Continued from our last, page 137--143.]

### INTERMITTENT FEVER.

THIS may be faid to be another variety of the endemic, in a milder degree, occafioned by the fame caufe, marfh-miafma, introduced in a fmaller proportion into a conftitution not difpofed to be affected with morbid movements. The cafes of this fever which occurred, happened in the healthy feafon of the year; they were moftly tertians. After an emetic and purgative had been been given, the bark was ordered to be taken every hour during the intermiffion. On the approach of the cold fit, 40 drops of antimonial wine, with 20 of tinct. opii. were adminiftered; which leffened the violence, and fhortened the duration of the paroxifm. In fome cafes, I found it beneficial to give wine and powerful ftimulants, fo as to keep up and increafe the action of the arterial fyftem, beyond the ufual period of the returning paroxyfm; while the bark was given every hour without intermiffion, unlefs prevented by the patient being afleep: careful centinels were placed over the fick, and they were frequently vifited in the night, by myfelf, or my affiftants. Calomel, in fmall dofes, was of benefit in fome cafes, where I conceived the diforder proceeded from vifceral obftruction; while others required a change of air, of which they had the benefit, when removed to Port-Royal Hofpital.

### DYSENTERY.

This difeafe is not limitted or peculiar to any climate; nor is there any natural caufe known to produce it: if it were occafioned by any particular quality in the air, the natives, as well as feamen and foldiers, would be attacked with it; but we find this is not the cafe, for when the dyfentery was raging in the Britifh army at the Cape of Good Hope, where great numbers died, not one of the inhabitants were feized with it; nor is it a difeafe known among them. Whenever it becomes epidemic among the inhabitants of any country, it may always be traced to infection introduced; it being the conftant attendant on camps, and the fcourge of an army, more deftructive than any other enemy.—I therefore confider it an *artificial* difeafe, brought on by thofe unavoidable hardfhips, to which foldiers and feamen are expofed. Cold and dampnefs, when the body is not fufficiently covered, by obftructing perfpiration, and increafing the determination of the fluids to the inteftines, fometimes combined with febrile miafma, produce the whole phenomena of dyfentery.

In

In the treatment of this disease, I generally began with an emetic of ipecacuanha, with a grain of tart. antimon. Bleeding was never employed, unless the patient was of a strong plethoric habit, and the febrile symptoms run high. Purges of salts, or rhubarb with calomel, were frequently repeated; at night, 40 drops of antimonial wine, with 20 of tinct. opii. which promoted perspiration, and relieved the spasms, when the tenesmus was troublesome. Emollient injections with opium, and fomentations were of use, when the pains were wandering; but when the pain became fixed in any part of the intestines, indicating inflammation, a large blister was instantly applied, which in every instance removed it; perspiration was kept up by repeated doses, either of preparations of antimony, or Dover's powder, assisted by warm diluents. In cases combined with the remittent symptoms, inducing great debility, accompanied with sanguinary discharges, bark and wine were given, with farinaceous vegetables for food, such as rice, sago, or tapioca; sometimes to ease pain, and procure a little rest, opium was given, but seldom without some medicine to determine its action to the surface.

A few cases occurred unattended with fever, violent gripings, or tenesmus: in these, mercurial purges and calomel, in small doses, were prescribed with success.

DIARRHŒA.

This disease generally arose from relaxation, brought on by eating unripe fruit, and committing other irregularities. It was easily removed by lenient purgatives, followed by astringents and tonics.

HEPATIC COMPLAINTS.

Having served in India with his Majesty's troops, during the campaigns of 1782, 1783, and 1784, in the Carnatic and Southern provinces, I had frequent opportunities of seeing liver-complaints, a disease often occurring in every hot climate, though not always suspected or discovered, and sometimes confounded with complaints of the pleura or lungs. The cases which occurred on board the Astrea were brought on by violent exercise in the sun, joined to the abuse of spirits. The patients complained of pain in the side, with some difficulty in respiration; the pulse full and frequent, sometimes they felt a pain in the shoulder, and about the region of the liver, which last, when pressed, was attended with a catching and troublesome cough. Bleeding, calomel-purges, and a blister applied to the side, and sometimes mercury in small doses, were alternately resorted to, until health was perfectly restored.

In chronic cases, the patients did not complain of acute pain, but a dull, obtuse pain in the hepatic region; the countenance dejected, and the animal spirits greatly diminished; the eyes dull, but not yellow; the tongue dry; the stools small, frequent, and sometimes mixed with mucus, as in dysentery; the the appetite irregular, often voracious. In these cases, without any preparation, I had recourse to mercury, either internally, or by friction, which last I preferred. The good effect of this treatment became obvious from the favourable change of that dejected countenance, so characteristic of the complaint, for a more cheerful one: and from the energy being restored to the hepatic system, which we discovered by the secretions; the stools becoming more regular, and of a natural colour: their diet consisted chiefly of small quantities of animal food, with the farinaceous vegetables.

SPASMODIC AFFECTIONS.

Those affections were mostly confined to the abdominal viscera, and brought on by lying upon deck in the night. The patients complained of excruciating pain and stricture, commonly about the umbilical region, nausea, and sometimes vomiting. If fomentations did not cure the pain, a large blister was applied; calomel with jallap taken internally, and glysters thrown up, until stools were procured, which removed the complaint.

One case occurred where the spasms were general. A man of a weak, irritable constitution, after taking an antimonial emetic, which operated rather violently, was seized with coldness on the surface of the whole body; violent contractions of the lower extremities, extending to the muscles of the abdomen, diaphragm, and ribs; the muscles of the face and neck became violently convulsed and distorted; the eyes sunk, and no pulse any where to be felt; respiration hardly to be perceived; he lay for some minutes in this state, so that, while the warm bath was getting ready, the by-standers, from his external appearance, concluded he was dead. Volatile and stimulating applications were used, together with frictions with hot cloths; injections of warm water and laudanum were thrown up; wine mulled with spices was given, until the heat of the body was restored, by persevering in these remedies. The disease at length yielded to this treatment; but he continued in a state of debility for some time, which rendered the administration of bark, cordials, and, at the same time, a nourishing diet, useful and necessary.

SCURVY.

The four cases of scurvy which occurred, were those of men who were devoted to irregular habits, and had for some years manifested a predisposition to the disease. Every care was taken to prevent it, by a sufficient allowance of vegetable aliment, a deficiency of which only can produce the disease; and before we went on a cruise, I took care to provide for the remedy on board. Plenty of lemon or lime-juice was given on the first symptom of the disorder, joined to what vegetable diet the ship afforded.

(To be concluded in our next.)

Experi-

